# Effects of donor killer-cell immunoglobulin-like receptor genotypes on clinical outcome after allogeneic hematopoietic stem cell transplantation—a systematic review and meta-analysis

**DOI:** 10.3389/fimmu.2026.1856878

**Published:** 2026-07-14

**Authors:** Yarui Huang, Qingrong Li, Xin Xu, Ju Li, Yan Zhu, Chengxin Luo, Jiegang Xu, Jiaming Liu, Jianmin Zhang, Ping Wang, Ya Tan, Yaqun Ding, Shuangnian Xu, Run Chen, Ling Wei

**Affiliations:** Center for Hematology, Southwest Hospital, Third Military Medical University, Chongqing, China

**Keywords:** allo-HSCT, clinical outcome, genotype, killer-cell immunoglobulin-like receptor, meta-analysis

## Abstract

**Purposed:**

Natural Killer cell kinetics and Killer-cell immunoglobulin-like receptors (KIRs) are increasingly recognized for their role during allogeneic hematopoietic stem cell transplantation (allo-HSCT), but the prognostic impact of donor KIR haplotype configurations on patient survival remains controversial. To clarify this relationship, we conducted a systematic review and meta-analysis evaluating the impact of donor KIR haplotypes on the survival of patients undergoing allo-HSCT.

**Methods:**

We systematically searched PubMed, Embase, the Cochrane Library, and CBM. Data were analyzed using RevMan5.4. Pooled hazard ratio (HR) for time-to-event data. Subgroup analyses were performed for sample size, area, donor type, T cell replete or deplete, GVHD prophylaxis, and lymphoid or myeloid.

**Results:**

17 eligible studies were included, and meta-analysis showed that donor KIR B/X genotype is associated with significantly superior overall survival (HR = 0.68, 95%CI, 0.56-0.82; P<0.0001), reduced relapse incidence (HR = 0.61, 95%CI, 0.44-0.85; P = 0.003), and improved relapse-free survival (HR = 0.61, 95%CI, 0.46-0.81; P = 0.0008).

**Discussion:**

The findings suggest that donor KIR B/X haplotype is associated with favorable survival outcomes in allo-HSCT including HLA-matched sibling, unrelated donor, and haploidentical transplantation, supporting its potential utility as a reference biomarker for donor selection.

## 1 Introduction

Allogeneic (1) hematopoietic stem cell transplantation (allo-HSCT) is a curative intervention applicable to a variety of diseases, especially hematological malignancies. Nevertheless, overall survival (OS) of patients receiving allo-HSCT remains suboptimal due to relapse and death from transplant-related complications including graft-versus-host disease (GVHD) and infections, etc (1, 2). Strategies for the prevention of relapse and GVHD have emerged as a critical focus in contemporary therapeutic research to improve OS after allo-HSCT.

Human leukocyte antigen (HLA) is a decisive factor for the choice of donor and eventual success of allo-HSCT, but accumulating evidence has established the prognostic significance of other non-HLA variables in HSCT, including donor age, ABO compatibility, and donor-specific antibody, necessitating their systematic evaluation into modern graft optimization protocols (3–5). Building on these findings, killer-cell immunoglobulin-like receptors (KIRs), which comprise inhibitory and activating subtypes and predominantly localized on the surface of NK cells, are increasingly recognized for their role in allo-HSCT (6). In humans, NK cell surveillance of classical HLA class I expression is primarily mediated by KIRs (7, 8). In allo-HSCT, NK cells, via their KIRs which recognize donor or recipient Major histocompatibility complex (MHC) class I molecules, modulate activation thresholds to mediate graft-versus-leukemia (GVL) effects, balance cytotoxicity to mitigate GVHD, and facilitate post-transplant immune reconstitution.

Historically, three models were developed in an attempt to improve donor selection for HSCT based on KIRs. The first model, termed donor-recipient KIR ligand-ligand model, is exclusively based on the HLA phenotype of the donor and recipient. This model is defined by KIR ligand incompatibility in the graft-vs-host (GVH) direction because the recipient lacks the donor class I allele group(s) which is recognized by KIRs. Crucially, this incompatibility paradigm was associated with reduced relapse rates (RR) and prevented GVHD incidence in acute myeloid leukemia (AML) patients undergoing HSCT (9, 10). Subsequently, Leung et al. (11) proposed the receptor-ligand model (or “missing KIR-ligand” model), which prioritizes donor KIR profiles over donor HLA. Notably, their analysis demonstrated that this model outperformed the ligand-ligand model in predicting primary disease relapse risk, establishing its superior prognostic utility for relapse prevention strategies in HSCT.

Lately, with a deeper understanding of KIR genes, and according to the specific KIR gene locus on the chromosome, a centromeric and telomeric KIR haplotype and genotype are further determined (12, 13). After assessing genotypes for the centromeric and telomeric parts of KIR locus, another NK alloreactivity model was used to analyze and compare the KIR genotypes of different donors. Diverse KIR haplotypes can be categorized into two separate biological groups: AA and B/X. KIR haplotypes are broadly categorized into AA and B/x types based on gene content, although this binary classification does not capture allele-level functional diversity (e.g., the differential ligand specificity of KIR2DL1*003 versus *004). Despite this simplification, this classification remains the most practical approach for population-level meta-analysis, as most published studies to date have used low-resolution genotyping methods (Polymerase Chain Reaction-sequence-specific primers or Polymerase Chain Reaction-sequence-specific oligonucleotide).

Several clinical studies have explored the prognostic value of donor KIR haplotypes in allo-HSCT. Heatley et al. (7) demonstrated that donor KIR B haplotype was linked to superior OS and a lower incidence of grade II–IV aGVHD. Bachanova et al. (14) further indicated that KIR B/x donors were associated with reduced relapse risk and favorable progression-free survival, while no obvious correlation was observed between donor KIR genotype and GVHD occurrence. Nevertheless, Zhou et al. (15) found no significant differences in OS, GVHD incidence, or RR between recipients transplanted from KIR AA and KIR B/X donors. Given these inconsistent observations regarding donor KIR haplotypes, It is necessary to further elucidate the potential clinical relevance of KIR haplotypes in allo-HSCT.

Here, we conducted a systematic meta-analysis evaluating the association between donor KIR haplotypes and clinical outcomes in allo-HSCT by synthesizing available evidence. This integrated approach provides an evidence-based framework to optimize donor selection protocols.

## 2 Materials and methods

### 2.1 Search strategy

This systematic review and meta-analysis were designed according to the Cochrane Handbook of systematic reviews and followed the Preferred Reporting Items for Systematic Reviews and Meta-analysis guidelines (16). We searched PubMed, the Cochrane Library, Embase, and CBM databases from inception April 27, 2025. A comprehensive search strategy encompassing PubMed, Embase, Cochrane Library, and CBM databases was executed through April 27, 2025, employing tailored search strings combining Medical Subject Headings (MeSH) terms and possible keywords specific to each database’s requirements. Keywords and Medical Subject Headings terms included Killer Inhibitory Receptors, KIR, hematopoi*, transplant*, graft*. Full search strategies are presented in **Supplementary Table 1**. Moreover, we did an extensive manual search through the references of the included articles to retrieve any missed papers.

### 2.2 Study selection

Eligible studies had to satisfy the following criteria (1): retrospective or prospective case-control or cohort studies and clinical trials reporting on donor KIR genotypes after allo-HSCT (2); availability or possibility to estimate data on hazard ratio (HR) with 95% CIs for OS, aGVHD, chronic GVHD (cGVHD), RR, disease-free survival (DFS).

The exclusion criteria included (1) results reported by a review, case study, comment, or conference abstract (2); animal studies only (3); duplicate publications from overlapping cohorts (4); ongoing/unpublished trials lacking extractable outcome data at database closure.

Titles and abstracts of all identified articles were separately screened by two reviewers (YR Huang and QR Li). Abstracts with unclear information were included for full-text review. In both stages, the senior author (Ling Wei) was consulted to resolve any conflicts in the decisions.

### 2.3 Definition of outcomes

Overall survival (OS) was defined as the time from transplantation until death from any cause. Relapse was defined as morphologic or clinical evidence of recurrence in the peripheral blood, bone marrow or extramedullary sites. Disease-free survival (DFS) was defined as the time elapsed between documentation of CR and either evidence of disease progression or death from any cause. Relapse-free survival (RFS) was defined as the time to relapse. Acute GVHD (aGVHD) was defined as development of any grade GVHD during the first 100 days post-transplantation; and chronic GVHD (cGVHD) was defined as GVHD at 100 days post-transplantation.

### 2.4 Data extraction

According to the inclusion and exclusion criteria, data were carefully extracted from all eligible studies independently by two reviewers, including the author, country, publication year, publication type, study design, the number of participants, patient characteristics, recruitment period, donor type, GVHD prophylaxis, and conditioning regimen. The clinical outcomes were as follows: OS, RR, DFS, aGVHD, cGVHD. For these outcomes, HRs and corresponding 95% CIs are extracted or estimated with the methods previously established by Tierney et al. (17).

### 2.5 Quality assessment

We utilized the Newcastle-Ottawa Quality Assessment Scale (NOS) tool to assess bias in the included studies (18). This tool evaluates selection, comparability, and outcome. The full score is 9 points, and a high score represents high methodological quality. The risk of bias was independently assessed by two reviewers, and any disagreements were resolved by a third reviewer.

### 2.6 Statistical analysis

All analyses were conducted in Review Manager Version 5.4. We merged the log HRs and corresponding 95% CIs of each study using the generic inverse-variance method for time-to-event data. The heterogeneity across studies was evaluated with the chi-square-based Cochran’s Q-test (P ≤ 0.10 was considered as significant evidence of heterogeneity) and the I^2^ statistic (I^2^ = 0-25%: no heterogeneity; I^2^ = 25-50%: low heterogeneity; I^2^ = 50-75%: moderate heterogeneity; I^2^ = 75-100%: large heterogeneity). A fixed-effect model was chosen for summary estimation if heterogeneity was not significant, whereas the random-effects model was used if heterogeneity was significant. Publication bias was assessed using funnel plots.

## 3 Results

### 3.1 Study inclusion

**Figure 1:**
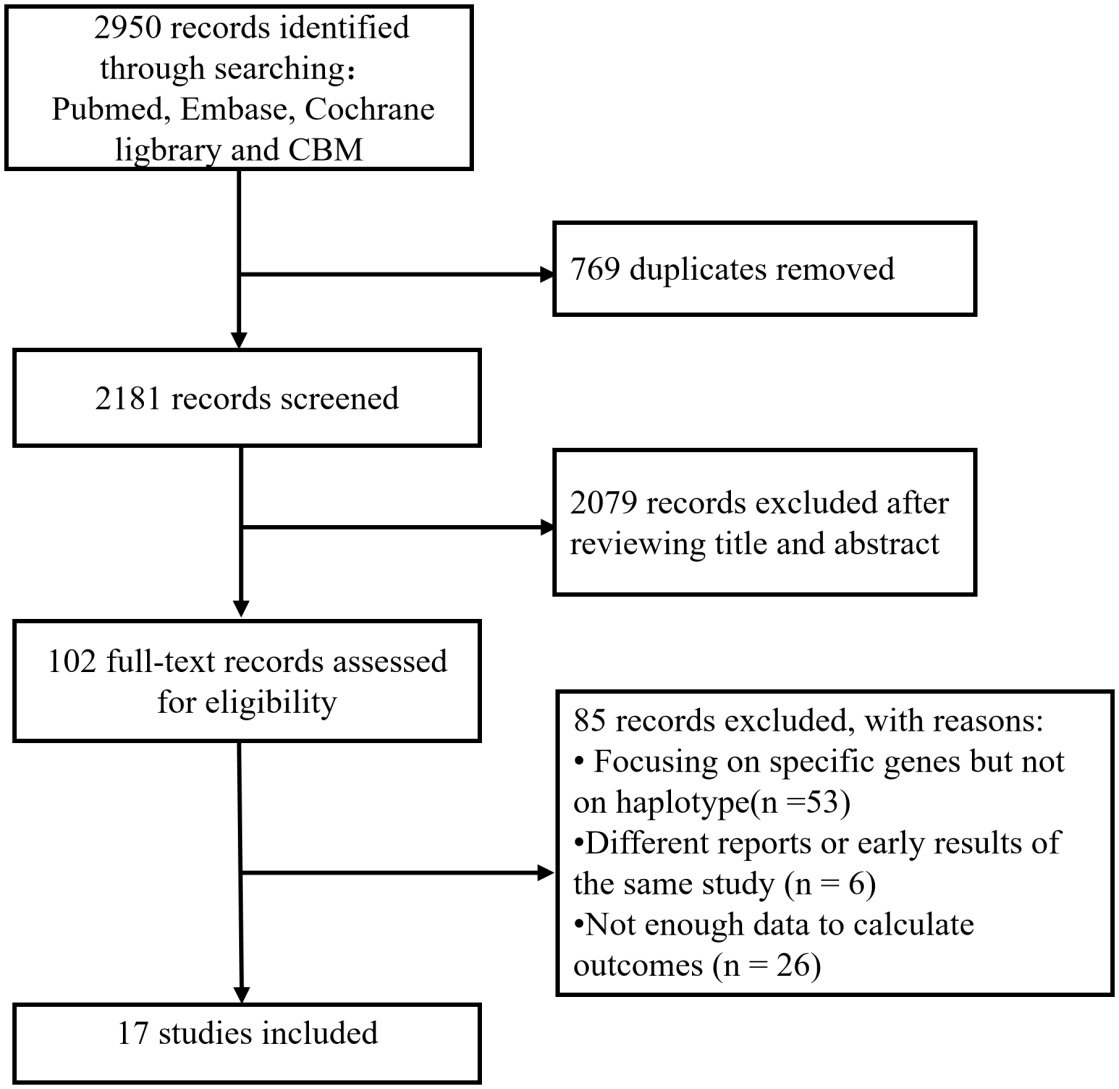
The flow chart of study selection.

The initial search of the four databases yielded 2950 records, of which 769 were removed as duplicates or irrelevant. After filtering the titles and abstracts, 2181 studies were eliminated because they were reviews, case studies, comments, or meeting minutes. After reading the full text of the remaining 102 articles, 85 were excluded due to the following reasons (1): failed to obtain available data (2); duplicate reports of the same study (3); focusing on specific genes but not on haplotype. Ultimately, 17 studies were included in our systematic review and meta-analysis (**Figure 1**).

### 3.2 Study characteristics

The characteristics of the included studies are shown in **Table 1**. All 17 studies were published between 2010 and 2022. Among them, 15 were retrospective studies and 2 were prospective studies. The citations were from 12 countries: 3 studies from US, 1 from Japan, 1 from Italy, 2 from UK, 2 from China, 1 from Turkey, 1 from Egypt, 2 from Spain,1 from Australia, 1 from Korea and 2 studies from Germany. 6 studies involved unrelated donors, 5 involved matched related donors, 2 involved both of unrelated donors and matched related donors, 3 involved matched related donors and haploidentical donors, 1 involved matched related donors, unrelated donors and mismatched family donors. Among them, 11 studies reported OS, 9 studies reported RR, 5 studies reported RFS, 6 studies reported grades II-IV aGVHD, 3 studies reported any grade aGVHD, and 5 studies reported cGVHD. The quality assessment scores ranged from 6 to 8, and the detailed score items for the 17 included studies are shown in **Table 2**.

**Table 1:** Characteristics of included studies.

Author	Publication year	Publication type	Country	Study design	Recruitment period	No.of patients	Gender (M/F)	Age(median,range; years)	Donor type	Conditioning	GVHD prophylaxis	T cell-replete or deplete	Lymphoid or myeloid	KIR genotyping method	NOS score
Bachanova et al.	2016	Article	US	retrospective	1990-2009	396	NA	50 (19–72)	URD	MAC/RIC	CsA/Tac	T cell–replete	Lymphoid	NA	8
Bao et al.	2015	Article	China	retrospective	2011-2014	210	127/83	28 (8–59)	URD	MAC	ATG-based	NA	Both	PCR-SSP	8
Bultitude et al.	2020	Article	UK	retrospective	1996-2011	119	73/46	NA	URD	MAC	NA	NA	Myeloid	PCR-SSP	6
Cooley et al.	2009	Article	UK	retrospective	1988-2003	448	274/174	NA	URD	MAC	NA	T cell–replete	Myeloid	SNP-based	7
Diaz et al.	2016	Article	Spain	retrospective	2005-2013	70	48/27	9 (0.5-19)	MRD/Haplo	RIC	CSP	T cell–deplete	Both	PCR	7
Elfishawi et al.	2017	Article	Egypt	retrospective	2010-2014	65	44/21	29 (8–51)	MRD	MAC	CSP+ methotrexate	NA	Both+SAA	PCR-SSO	8
Heatley et al.	2018	Article	Australia	retrospective	2002-2007	145	NA	NA	MRD	MAC/RIC	CsA	T cell–deplete	Both+others	PCR-SSP	6
Hong et al.	2021	Article	US	prospective	2000-2013	81	40/41	NA	MRD	MAC/RIC	CSP+ methotrexate/mycophenolate	T cell–replete	Myeloid	PCR-SSO	7
Hosokai et al.	2017	Article	Japan	prospective	1989-2011	106	55/51	NA	MRD/URD/MMFD	MAC/RIC	CSP/Tac	T cell–replete	Both	PCR-SSO	8
Kröger et al.	2011	Letter	Germany	retrospective	1997-2008	118	43/75	51 (29–68)	MRD/URD	MAC/RIC	ATG-based	NA	Multiple myeloma	PCR-SSO	6
Littera et al.	2010	Article	Italy	retrospective	1993-2007	78	NA	10(1-29)	URD	RIC	CsA	NA	Thalassaemia	NA	7
Oevermann et al.	2014	Brief report	Germany	retrospective	1996-2013	85	NA	NA	MRD/Haplo	NA	NA	T cell–deplete	Lymphoid	NA	6
Park et al.	2015	Article	Korea	retrospective	2011-2013	59	34/25	41(18-65)	URD/MRD	MAC	ATG-based	NA	Both	PCR-SSO	6
Sahin et al.	2017	Article	Turkey	retrospective	1994-2014	96	50/46	NA	MRD	MAC/RIC	PTcy-based	T cell–replete	Myeloid	PCR-SSP	8
Torío et al.	2017	Article	Spain	retrospective	2013-2014	30	15/15	45(5-69)	MRD/Haplo	MAC/RIC	PTcy-based	T cell–replete	NA	PCR-SSP	7
Zhou et al.	2013	Article	China	retrospective	2005-2012	219	120/99	28(8-63)	MRD	MAC/RIC	CsA	NA	Both	PCR-SSP	8
Moyer et al.	2022	Article	US	retrospective	2008-2013	77	37/40	53(18-68)	URD	MAC/RIC	NA	NA	Myeloid	PCR-SSO	8

URD, unrelated donors; MRD, matched related donors; MMFD, mismatched family donors; Haplo, haploidentical; MAC, myeloablative conditioning; RIC, reduced intensity conditioning; CSP, cyclosporine; PTcy, post-transplant cyclophosphamide; ATG, anti-thymocyte globulin; CsA, cyclosporine A; TAC, tacrolimus; PCR, Polymerase Chain Reaction; SSO, sequence-specific oligonucleotide; SSP, sequence-specific primers; SNP, Single Nucleotide Polymorphism; NA, not applicable.

**Table 2:** The assessment of the risk of bias in each cohort study using the Newcastle-ottawa scale.

Study	Selection				Comparability		Outcome			Total
	A	B	C	D	E	F	G	H	I	
Bachanova et al.	1	1	1	1	1	1	0	1	1	8
Bao et al.	1	1	1	0	1	1	1	1	1	8
Bultitude et al.	1	1	1	0	1	0	1	1	1	7
Cooley et al.	1	1	1	0	1	1	0	1	1	7
Diaz et al.	1	1	1	0	1	1	0	1	1	7
Elfishawi et al.	1	1	1	0	1	1	1	1	1	8
Heatley et al.	1	1	0	0	1	1	1	1	1	7
Hong et al.	1	1	1	0	1	1	1	0	1	7
Hosokai et al.	1	1	1	0	1	1	1	1	1	8
Kröger et al.	1	1	1	0	1	0	0	1	1	6
Littera et al.	1	1	1	0	1	1	1	0	1	7
Oevermann et al.	1	1	1	0	1	0	0	1	1	6
Park et al.	1	1	1	0	1	1	0	1	1	7
Sahin et al.	1	1	1	1	1	0	1	1	1	8
Torío et al.	1	1	1	0	1	1	0	1	1	7
Zhou et al.	1	1	1	0	1	1	1	1	1	8
Moyer et al.	1	1	1	1	1	0	1	1	1	8

A=representativeness of the exposed cohort, B=selection of the non exposed cohort, C=ascertainment of exposure, D=demonstration that outcome of interest was not present at start of study, E =study controls for select the most important factor, F=study controls for any additional factor, G=Assessment of outcome, H=Was follow-up long enough for outcomes to occur, I=adequacy of follow up of cohorts. Primary outcome of Meta-analysis.

### 3.3 Outcomes

#### 3.3.1 Overall survival

**Figure 2:**
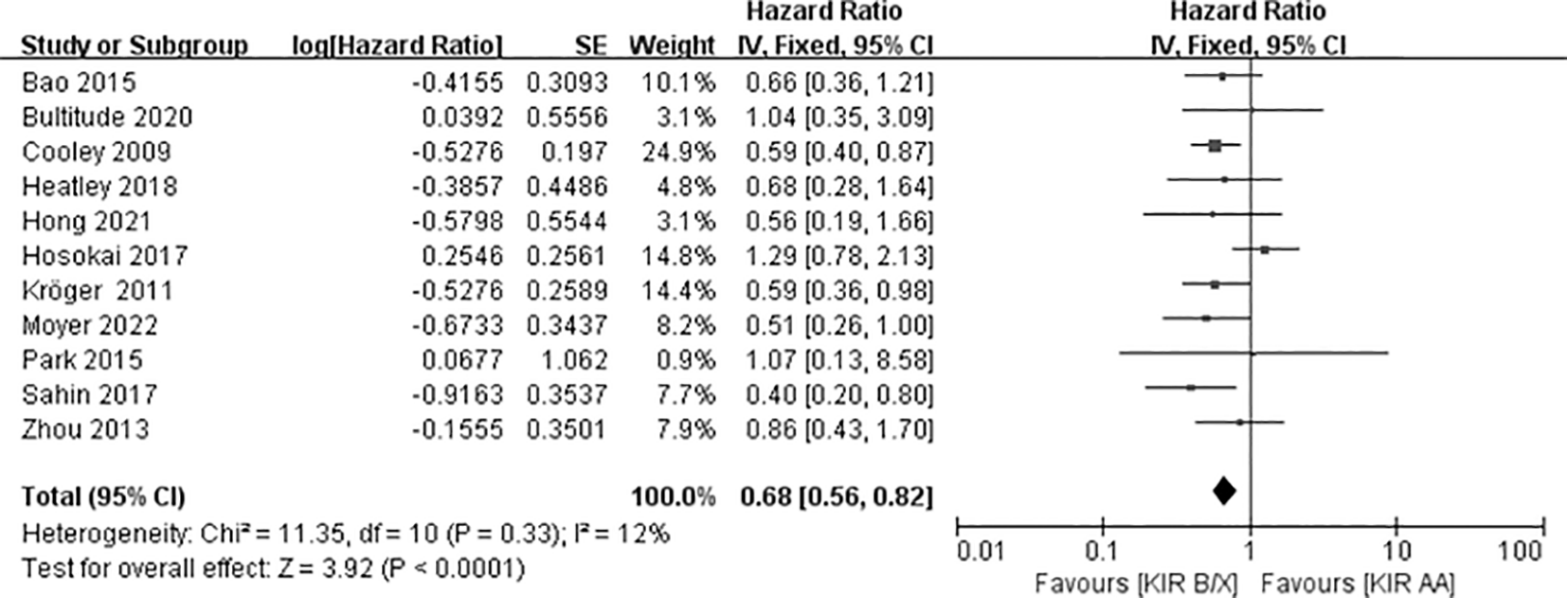
Meta-analysis of the association between donor KIR genotype and overall survival.

11 studies (1, 7, 15, 19–26) reported OS both in patients with donor KIR AA and donor KIR B/X and heterogeneity among the studies was low (I^2^ = 12%; P = 0.33). Therefore, a fixed-effect model was used. Our meta-analysis results showed that OS in donor KIR B/X group was significantly higher than that in donor KIR AA group (HR = 0.68, 95%CI, 0.56-0.82; P<0.0001; **Figure 2**). Sensitivity analyses for all outcomes indicated the robustness and reliability of the findings (**Table 3**). Subgroup analyses stratified by sample size, area, donor type, T-cell replete or deplete strategies, GVHD prophylaxis, and lymphoid or myeloid showed no significant interactions between these factors and the prognostic impact of donor KIR genotypes in allo-HSCT. However, the favorable effect of donor KIR B/X haplotype was not observed in eastern populations or under either T-cell replete or T-cell deplete conditions (**Supplementary Figure 1**).

**Table 3:** Sensitivity analysis of the included studies.

First author (year published)	Statistics with study removed				
	Points	Lower limit	Upper limit	Z-value	P-value
OS					
Bao.2015	0.68	0.56	0.948	3.68	0.0002
Bultitude.2020	0.67	0.55	0.82	3.99	<0.0001
Cooley.2009	0.71	0.57	0.89	2.98	0.003
Heatley.2018	0.68	0.56	0.82	3.92	<0.0001
Hong.2021	0.68	0.55	0.83	3.79	0.0001
Hosokai. 2017	0.61	0.49	0.75	4.66	<0.0001
Kröger.2011	0.70	0.57	0.86	3.40	0.0007
Moyer.2022	0.70	0.57	0.85	3.50	0.0005
Park.2015	0.68	0.56	0.82	3.94	<0.0001
Sahin.2017	0.71	0.58	0.87	3.33	0.009
Zhou.2013	0.67	0.55	0.82	3.95	<0.0001
aGVHD (II-IV)					
Bachanova.2016	0.98	0.55	1.76	0.07	0.94
Cooley.2009	0.98	0.657	1.69	0.08	0.94
Hosokai.2017	0.95	0.69	1.31	0.31	0.76
Littera.2010	1.11	0.88	1.39	0.86	0.39
Zhou.2013	1.07	0.69	1.66	0.31	0.75
RR					
Bachanova 2016	0.61	0.40	0.92	2.35	0.02
Bultitude.2020	0.59	0.41	0.84	2.87	0.004
Cooley.2009	0.61	0.40	0.93	2.29	0.02
Diaz.2016	0.67	0.53	0.84	3.40	0.0007
Elfishawi.2017	0.60	0.43	0.82	3.21	0.001
Hosokai.2017	0.57	0.41	0.79	3.39	0.0007
Oevermann.2014	0.65	0.46	0.92	2.43	0.02
Sahin.2017	0.63	0.44	0.89	2.58	0.010
Zhou.2013	0.60	0.42	0.86	2.79	0.005
RFS					
Bao.2015	0.60	0.44	0.81	3.29	0.0010
Cooley.2009	0.69	0.46	1.03	1.81	0.07
Heatley 2018	0.59	0.44	0.80	3.50	0.005
Hong.2021	0.67	0.47	0.89	2.69	0.007
Zhou 2013	0.56	0.40	0.77	3.53	0.004
cGVHD					
Bachanova.2016	1.83	0.97	3.45	1.88	0.06
Cooley.2009	1.51	0.46	5.00	0.68	0.50
Torío.2017	1.15	0.70	1.88	0.55	0.58

#### 3.3.2 Relapse

**Figure 3:**
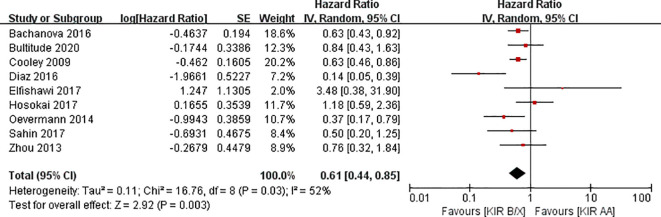
Meta-analysis of the association between donor KIR genotype and relapse.

9 studies (14, 15, 20, 21, 26–29) reported RR both in donor KIR B/X and KIR AA groups. There was significant heterogeneity among the studies, so a random-effect model was adopted (I^2^ = 52%; P = 0.03). Our meta-analysis results showed that RR in donor KIR B/X group was significantly lower than donor KIR AA group (HR = 0.61, 95%CI, 0.44-0.85; P = 0.003; **Figure 3**). Sensitivity analyses confirmed low heterogeneity with individual exclusion of each of the 9 studies (**Table 3**). Subgroup analyses for relapse risk according to based on sample size, area, donor type, T cell replete or deplete, and lymphoid or myeloid, the results demonstrated no significant interaction between these factors and the predictive effect of donor KIR genotypes for RR (**Supplementary Figure 2**).

#### 3.3.3 Relapse-free survival

**Figure 4:**
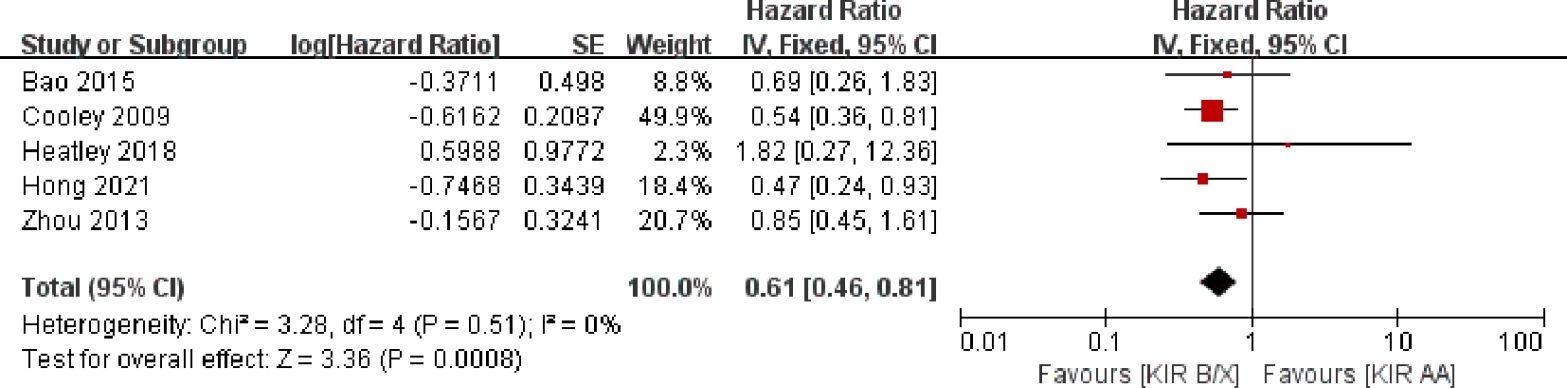
Meta-analysis of the association between donor KIR genotype and relapse-free survival.

5 studies (7, 15, 19, 21, 22) compared the RFS between two groups. There was no heterogeneity among the studies, so a fixed-effect model was adopted (I^2^ = 0%; P = 0.51). Meta-analysis results showed that there was no difference between two groups (HR = 0.61, 95%CI, 0.46-0.81; P = 0.0008; **Figure 4**). Sensitivity analysis showed low statistical heterogeneity of the meta-analysis (**Table 3**). Further subgroup analyses for RFS according to sample size, area and donor type demonstrated that these factors exerted no modifying influence on the overall prognostic performance of donor KIR genotype (**Supplementary Figure 3**).

#### 3.3.4 aGVHD

**Figure 5:**
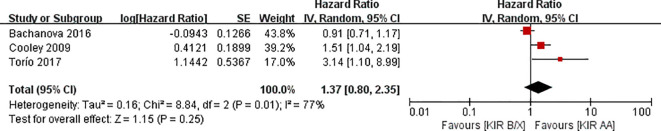
Meta-analysis of the association between donor KIR genotype and acute graft-versus-host disease.

5 studies (14, 15, 23, 30, 31) provided sufficient information on grades II-IV aGVHD and heterogeneity among the studies was significant, so a random-effect model was adopted (I^2^ = 56%; P = 0.06). Our meta-analysis indicated that there was no difference between donor KIR B/X group and donor KIR AA group (HR = 1.04, 95%CI, 0.73-1.47; P = 0.84; **Figure 5**). Excluding individual studies focusing on grade II-IV aGVHD in turn during sensitivity testing yielded stable pooled estimates with low heterogeneity (**Table 3**). Further subgrouping by sample size, area, and donor type revealed that these factors did not alter the association between donor KIR genotype and aGVHD risk (**Supplementary Figure 4**).

#### 3.3.5 cGVHD

**Figure 6:**
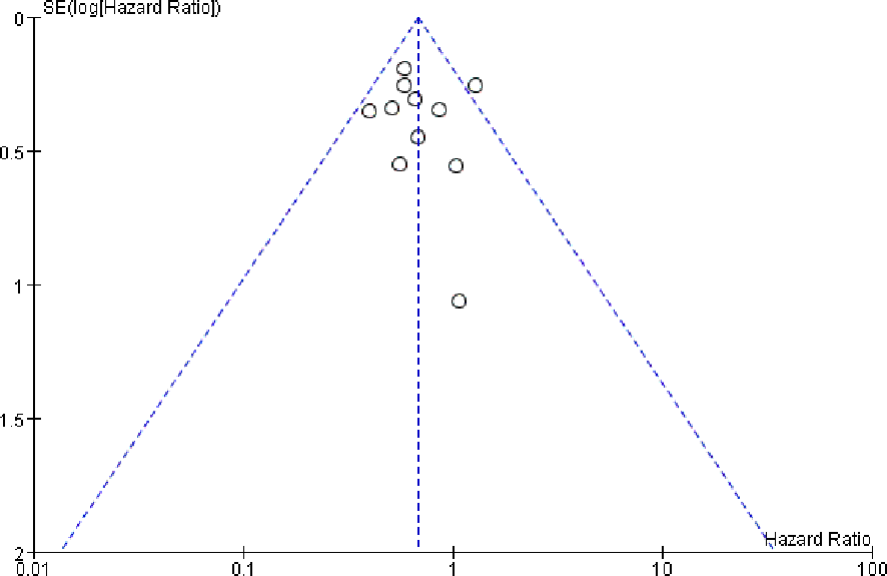
Meta-analysis of the association between donor KIR genotype and chronic graft-versus-host disease.

3 studies (14, 30, 32) reported data on cGVHD of patients after allo-HSCT. There was significant heterogeneity among the studies, so a random-effect model was adopted (I^2^ = 77%; P = 0.01). And our meta-analysis showed that there was no difference between donor KIR B/X group and donor KIR AA group (HR = 1.37, 95%CI, 0.80-3.25; P = 0.25; **Figure 6**). Sensitivity analysis showed low statistical heterogeneity of the meta-analysis after excluding each of 3 studies in turn (**Table 3**).

## 4 Publication bias

**Figure 7:**
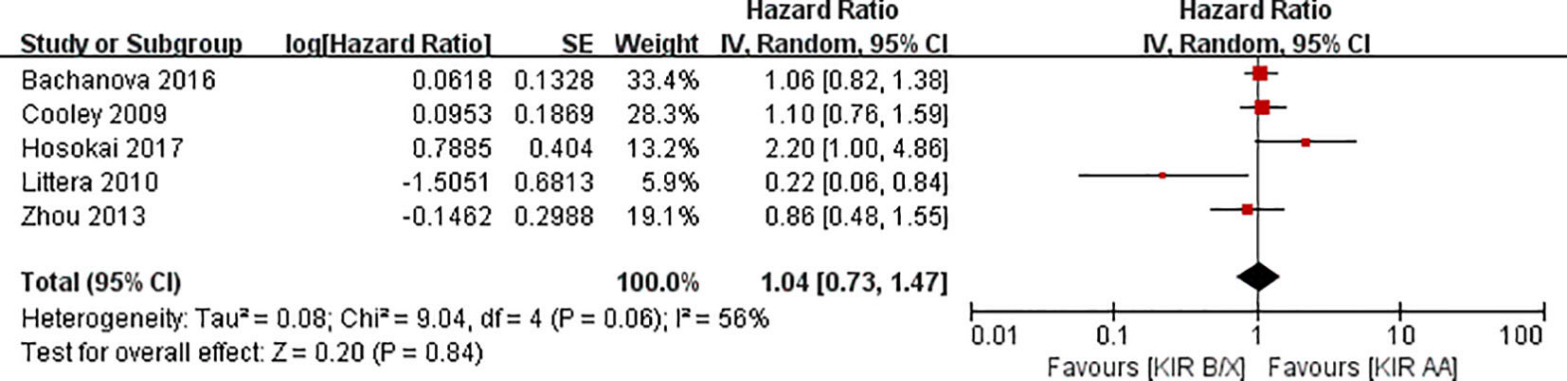
Funnel plot.

Publication bias was qualitatively examined using a funnel plot (**Figure 7**), and risk bias assessment analyses were conducted on the results that included at least 10 studies. Thus, funnel plots of OS were used to evaluate the publication bias. The funnel plots for the included studies in this meta-analysis of OS were not symmetrical, indicating that potential publication bias existed.

## 5 Discussion

HLA compatibility between donors and recipients remains a cornerstone of successful hematopoietic cell transplantation. Despite promising evidence for NK cells in relapse prevention, the reported anti-leukemic effects of KIR mismatch on transplantation outcomes show significant inconsistency (9). This variation may stem from differing definitions of KIR mismatch across studies, which encompass the ligand-ligand, receptor–ligand, and haplotype models. Consequently, studies evaluating the clinical impact of KIR incompatibility have yielded conflicting results. However, the KIR genotype of the donor, encoding receptors for these hyperpolymorphic HLA, is not routinely considered in allo-HSCT selection (20). Emerging evidence suggests that KIR genotypes demonstrate prognostic relevance in transplantation outcomes, though multicenter studies report discordant findings regarding their clinical impact.

Our systematic review and meta-analysis synthesizes evidence from 17 studies comprising more than 2,300 allo-HSCT recipients to evaluate the prognostic impact of donor KIR genotypes. Our findings demonstrate that grafts from donors carrying the KIR B/x haplotype confer significantly superior OS, reduced relapse incidence, and improved RFS compared with grafts from KIR AA donors. These results resolve longstanding controversies regarding the clinical utility of donor KIR genotyping and establish KIR B/x as a reference indicator for donor selection optimization. Subgroup analyses stratified by donor type further verified that this favorable prognostic effect was significant across multiple clinical transplantation modalities, including matched related donor, unrelated donor, and haploidentical allo-HSCT, covering the mainstream transplantation types in clinical practice. The survival advantage associated with KIR B/x donors is mainly rooted in enhanced NK cell–mediated GVL activity (33, 34). KIR B haplotypes are enriched for activating receptors such as KIR2DS1, KIR2DS2, and KIR3DS1, which potentiate “missing-self” recognition of NK cells against recipient’s residual malignant cells lacking cognate HLA class I ligands (6). Crucially, inhibitory receptors within the B haplotype exhibit high-affinity binding to HLA-C1 ligands, facilitating rigorous NK cell education and functional maturation, resulting in a repertoire with heightened cytotoxicity against leukemia blasts while preserving tissue tolerance (35).

Our analysis revealed no significant association between donor KIR genotype and overall acute or cGVHD incidence. This dissociation may stem from differences in the effector mechanisms driving GVL effects versus GVHD. The main mechanism of GVHD is that donor T cells recognize host alloantigens through direct TCR-peptide/MHC interactions, triggering inflammatory cytokine storms and tissue damage (36, 37). While KIR genotypes profoundly modulate NK cell alloreactivity against malignant cells through “missing-self” recognition, this cytotoxicity does not extend to non-hematopoietic tissues typically affected in GVHD (35). The education and licensing processes that calibrate NK cell responsiveness inherently enforce tolerance to healthy tissues including skin, liver, and gastrointestinal mucosa that express intact HLA class I molecules, rendering them incapable of initiating the pathogenic cascades that characterize clinical GVHD. In addition, the mechanism driving both aGVHD and cGVHD involves donor-derived alloreactive T cells. These cells recognize host alloantigens through direct TCR-peptide/MHC interactions, triggering inflammatory cytokine storms and tissue damage (36, 37). The pathogenesis of cGVHD represents a more complex process of immune dysregulation. Beyond the attack by donor alloreactive T cells on normal tissues, it involves thymic damage leading to central immune tolerance defects, abnormal activation of follicular helper T cells and B cells, autoantibody production, and ultimately culminates in fibrosis and sclerosis of multiple organs (38). While NK cells may infiltrate GVHD-affected organs, they likely serve regulatory roles, such as secreting immunomodulatory cytokines (IL-10, TGF-β) or eliminating host antigen-presenting cells, rather than driving tissue injury (39, 40). Furthermore, NK cells are the earliest lymphoid subset to reconstitute following HSCT, recovering at approximately day 30 (41). Nevertheless, their numerical dominance is transient, as T cell populations rapidly expand and soon become predominant. As such, NK cells possess only a narrow time window to exert any GVHD-associated alloreactivity. This capacity is further restricted by mandatory NK cell licensing and maturation, which intrinsically constrain their ability to initiate the pathological cascade of GVHD.

Building on these findings, we further verified the prognostic stability of KIR B/x genotype under the interference of common immunosuppressive regimens and different T cell strategies. Commonly used immmunosuppresive regimens and T cell replete/depleted grafts can affect NK cell reconstituion kinetics as previously reported (4). This emerging field of NK cell reconstitution is particularly important, as NK cells serve as the dominant lymphoid cell type in the post-transplant therapeutic window, and graft-derived T cells may further hinder NK cell recovery by competing for interleukin-15 (IL-15). To address this concern, we performed additional subgroup analyses stratified by anti-thymocyte globulin (ATG), post-transplant cyclophosphamide (PTCy) administration, as well as T cell-replete or T cell-deplete strategies. The results consistently demonstrated that the favorable prognostic impact of donor KIR B/x genotype was maintained not only in both ATG and PTCy subgroups, but also in both T cell-replete and T cell-deplete subgroups. This indicates that the survival benefit conferred by KIR B/x is independent of these immunosuppressive interventions and T cell manipulation strategies, further supporting its robust prognostic value. We fully recognize the importance of NK cell reconstitution kinetics and will focus more closely on its dynamic pattern in future prospective studies, further exploring the interplay among ATG/PTCy immunosuppression, T-cell-mediated IL-15 competition, T cell-replete/deplete strategies, KIR genotypic background, and long-term transplant outcomes to clarify the underlying mechanisms and provide more refined evidence for clinical practice.

Our meta-analysis clarifies several persistent controversies in the field. Unlike historical KIR–ligand incompatibility models requiring donor–recipient HLA mismatch, the donor-centric KIR B/x paradigm demonstrates efficacy even in HLA-matched settings, simplifying clinical implementation by eliminating recipient genotype dependency. Subgroup analyses indicated diminished KIR B/x effects in Asian populations, potentially attributable to high frequencies of non-functional KIR2DS4 variants in these cohorts, suggesting that population-specific KIR interpretation frameworks may be necessary (42, 43). Importantly, HLA matching remains the core and primary principle for donor selection in allogeneic hematopoietic stem cell transplantation (44). The KIR B/x haplotype serves only as an auxiliary reference index and should not take precedence over HLA compatibility. Current evidence is insufficient to demonstrate that the KIR B/x haplotype confers superior clinical value compared with conventional clinical factors, including donor age, sex, blood type, and CMV serostatus, nor does it support hierarchical ranking of these prognostic indicators (45). Based on our evidence, we propose that KIR B/x donors can be prioritized as a clinically valuable immunological indicator for donor selection under specific immunological backgrounds.

There are some limitations in our meta-analysis. First, most studies included were retrospective cohorts that may have had potential confounders, including heterogeneity in patients’ characteristics (such as age, gender, disease type), conditioning regimens, GVHD prophylaxis, donor-recipient KIR genotype mismatch, donor types. Second, it is with regret that we are unable to perform subgroup analyses since most of the included patients with different characteristics and receiving allo-HSCT in different settings without reporting subgroup results. Third, although they are all KIR B donors, the KIR genotype of the recipients will also affect the prognosis (35, 40). Nevertheless, the current published studies are not enough to be merged into our meta-analysis. In the future, we need to further confirm the influence of different combinations of donor and recipient KIR types on the prognosis. Fourth, we did not conduct further stratification based on refined centromeric and telomeric KIR subtypes. However, most included studies only reported conventional KIR B/x classification and lacked detailed genotyping data on high-resolution KIR subtyping and KIR B content grading, making further subgroup analysis unfeasible. Subsequent studies with detailed KIR haplotype typing are needed to address this issue. Fifth, conventional KIR genotyping methods (Polymerase Chain Reaction-sequence-specific primers or Polymerase Chain Reaction-sequence-specific oligonucleotide) adopted by included studies only detect the presence or absence of KIR genes, and fail to identify allele-level functional differences(e.g., the differential ligand specificity of KIR2DL1*003 versus *004, the functional difference between full-length and deleted KIR2DS4 variants, and the variable expression levels of KIR3DL1 alleles) (46–48). This low-resolution detection leads to technical heterogeneity, introduces analytical noise, and may weaken the true clinical effects of KIR genotypes. Fortunately, emerging next-generation sequencing-based approaches such as KIR probe interpretation allow direct and accurate KIR haplotype determination without statistical inference, which will improve the reliability and comparability of future relevant studies. Lastly, the impacts of KIR genes remain unclear because different studies reported conflicting results. For example, Cardozo et al. (36) showed that patients with donor KIR2DS2 undergoing HSCT had lower OS and event-free survival, whereas Neuchel et al. (35) reported that patients with donor KIR2DS2 had higher OS and DFS. However, it is impractical to investigate the differences between various KIR genes due to the fact that most included studies did not report relevant results. Further well-designed studies can be helpful to determine whether KIR genes impact survival outcomes in HSCT patients.

## 6 Conclusions

This meta-analysis reveals the potential prognostic implication of donor KIR B/x genotype in allo-HSCT including HLA-matched sibling, unrelated donor, and haploidentical transplantation. The KIR haplotype serves as a valuable population-level tool for understanding transplant immunology, guiding research design and informing clinical guidelines, while functioning as one of multiple reference indicators for individualized donor selection in clinical decision-making.

## Data Availability

The original contributions presented in the study are included in the article/**Supplementary Material**. Further inquiries can be directed to the corresponding authors.

## Supplementary material

Supplementary Figure 1 Meta-analysis of the association between donor KIR genotype and OS in subgroup analysis according to **(A)** sample size, **(B)** area, **(C)** donor type, **(D)** T cell replete or deplete, **(E)** anti-thymocyte globulin-based (ATG-based) or post-transplant cyclophosphamide-based (PTCy-based) and **(F)** Lymphoid or myeloid.

Supplementary Figure 2 Meta-analysis of the association between donor KIR genotype and RR in subgroup analysis according to **(A)** sample size, **(B)** area, **(C)** donor type, **(D)** T cell replete or deplete and (E) Lymphoid or myeloid.

Supplementary Figure 3 Meta-analysis of the association between donor KIR genotype and RFS in subgroup analysis according to **(A)** sample size, **(B)** area and **(C)** donor type.

Supplementary Figure 4 Meta-analysis of the association between donor KIR genotype and aGVHD in subgroup analysis according to **(A)** sample size and **(B)** area.
