# Supplementary material for: Effects of donor killer-cell immunoglobulin-like receptor genotypes on clinical outcome after allogeneic hematopoietic stem cell transplantation—a systematic review and meta-analysis
Source: Front Immunol. 2026 Jul 14;17:1856878. doi: 10.3389/fimmu.2026.1856878 (PMC13407179; doi:10.3389/fimmu.2026.1856878)
Supplement: Supplementary Figure 1 — Meta-analysis of the association between donor KIR genotype and OS in subgroup analysis according to (A) sample size, (B) area, (C) donor type, (D) T cell replete or deplete, (E) anti-thymocyte globulin-based (ATG-based) or post-transplant cyclophosphamide-based (PTCy-based) and (F) Lymphoid or myeloid. [file DataSheet1.docx]

| Search number | Query | Results |
| --- | --- | --- |
| 14 | #12 AND #13 | 708 |
| 13 | #1 OR #2 OR #3 OR #4 OR #5 OR #6 OR #7 | 6,726 |
| 12 | #11 AND #10 | 147,661 |
| 11 | (hematopoi*) OR (bone marrow) | 392,667 |
| 10 | (((((((((((((transplant*) OR (graft*)) OR (allotransplant*)) OR (allo-transplant)) OR (homotransplant*)) OR (homo-transplant*)) OR (retransplant*)) OR (re-transplant*)) OR (autotransplant*)) OR (auto-transplant*)) OR (allograft*)) OR (allo-graft*)) OR (homograft*)) OR (homo-graft*) | 1,058,226 |
| 9 | Bone marrow | 292,876 |
| 8 | hematopoi* | 161,631 |
| 7 | (Killer Immunoglobulin-Like Receptor) OR (Killer Immunoglobulin Like Receptor) | 4,052 |
| 6 | KIR Family Receptors | 3,434 |
| 5 | (Killer Cell Immunoglobulin-Like Receptors) OR (Killer Cell Immunoglobulin Like Receptors) | 3,873 |
| 4 | (Killer Cell Immunoglobulin Like Receptor) OR (Killer Cell Immunoglobulin-Like Receptor) | 4,002 |
| 3 | Killer Inhibitory Receptor | 5,459 |
| 2 | Killer Inhibitory Receptors | 5,733 |
| 1 | "Receptors, KIR"[Mesh] | 2,596 |

**Supplementary Table 1 Full search terms**

**
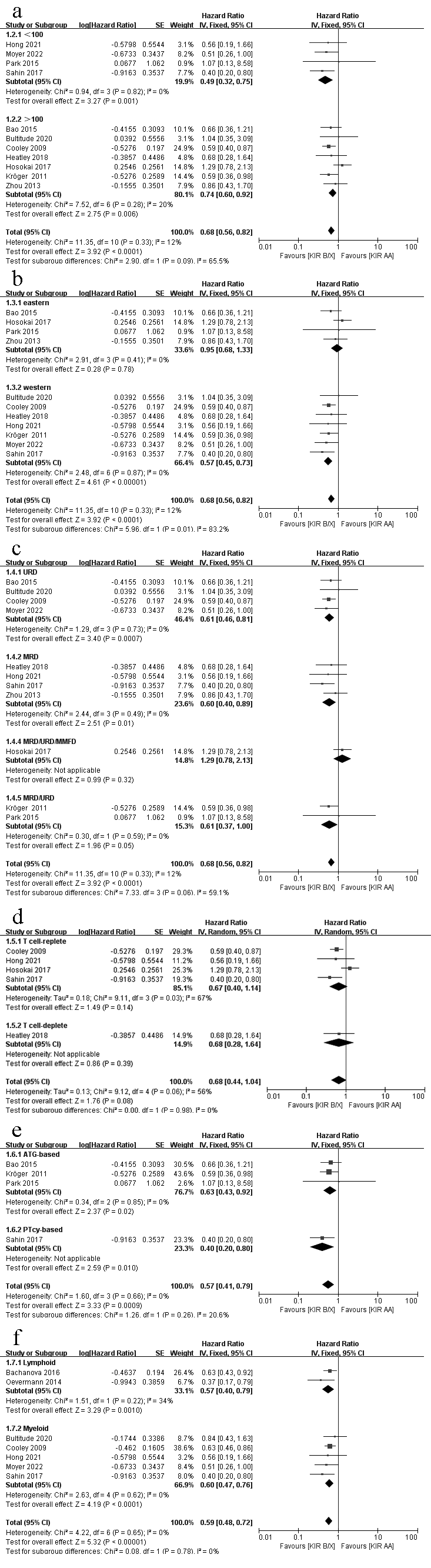
**

**Supplementary Figure 1. Meta-analysis of the association between donor KIR genotype and overall survival in subgroup analysis according to (a) sample size, (b) area, (c) donor type , (d) T cell replete or deplete, (e) anti-thymocyte globulin-based (ATG-based) or post-transplant cyclophosphamide-based (PTCy-based) and (f) Lymphoid or myeloid.**

**
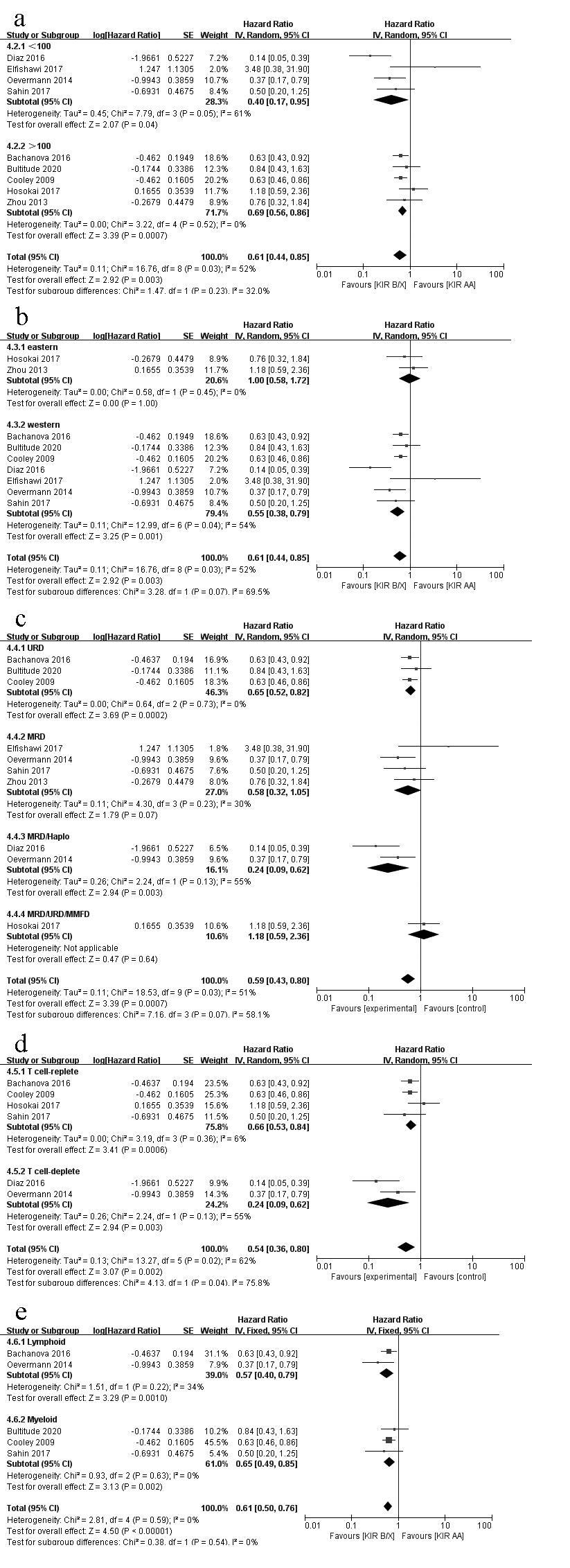
**

**Supplementary Figure 2. Meta-analysis of the association between donor KIR genotype and relapse in subgroup analysis according to (a) sample size, (b) area, (c) donor type, (d) T cell replete or deplete and (e) Lymphoid or myeloid.**

**
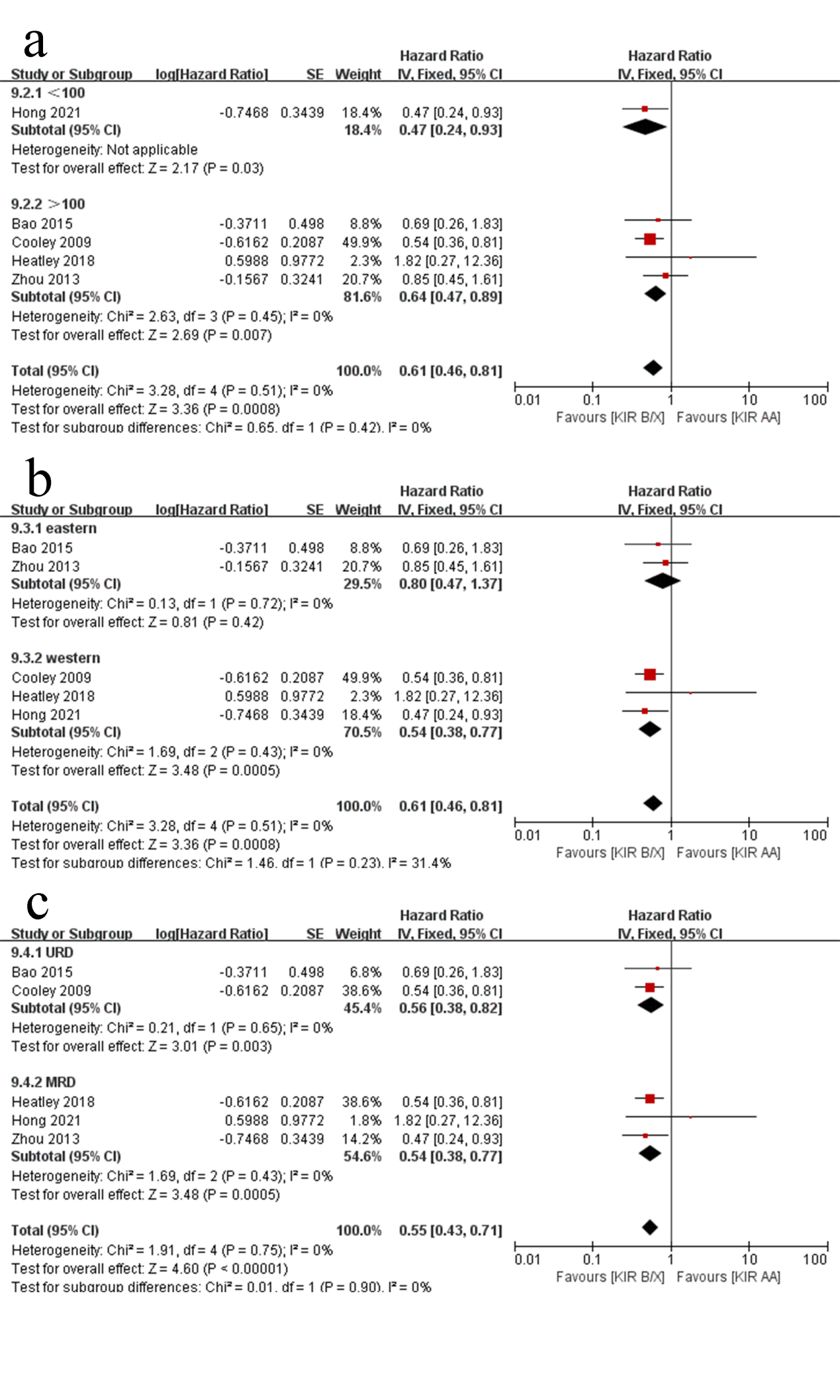
**

**Supplementary Figure 3. Meta-analysis of the association between donor KIR genotype and relapse-free survival in subgroup analysis according to (a) sample size, (b) area and (c) donor type.**

**
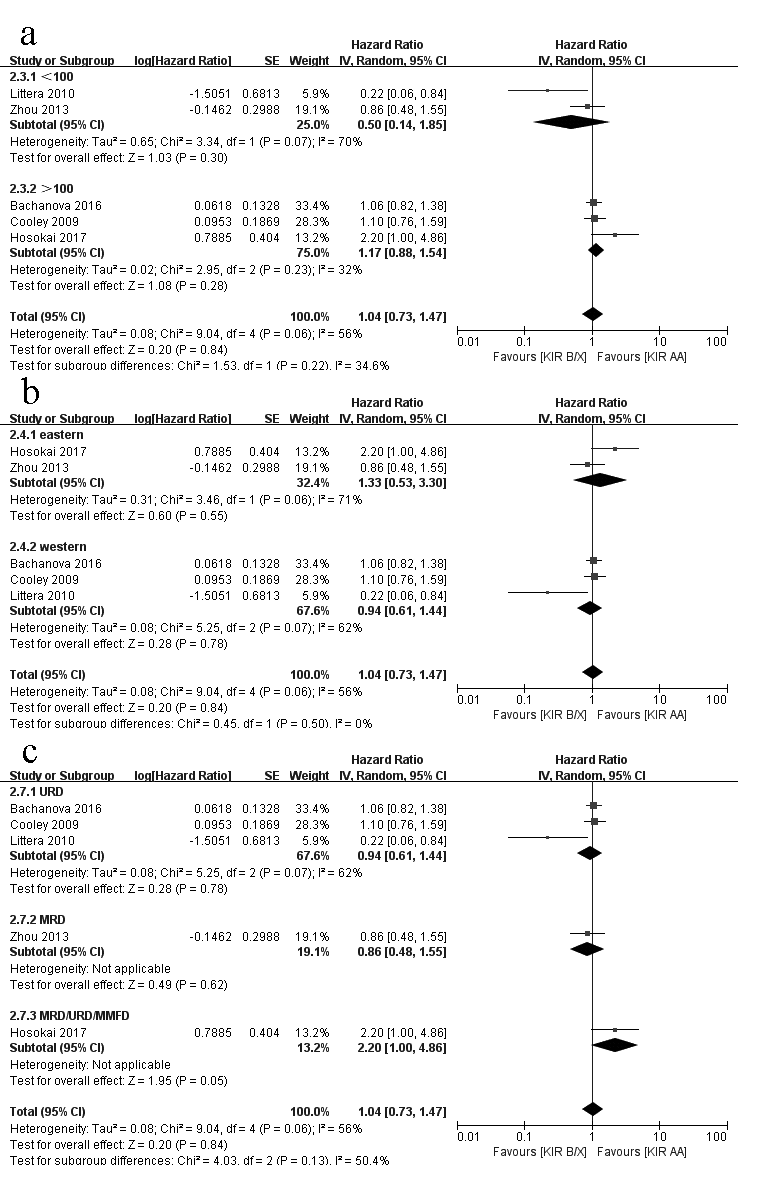
**

**Supplementary Figure 4. Meta-analysis of the association between donor KIR genotype and acute graft-versus-host disease in subgroup analysis according to (a) sample size and (b) area.**
